# QTc prolongation in Black diabetic subjects with cardiac autonomic neuropathy

**DOI:** 10.4314/ahs.v17i4.17

**Published:** 2017-12

**Authors:** Ogba J Ukpabi, Basden JC Onwubere

**Affiliations:** 1 Federal Medical Centre, Umuahia, Nigeria; 2 University of Nigeria Teaching Hospital, Ituku Ozalla, Enugu, Nigeria

**Keywords:** QTc prolongation, Cardiac Autonomic Neuropathy, Black diabetics, sudden cardiac death

## Abstract

**Background:**

Prolonged corrected QT (QTc) has been identified as a risk factor for malignant arrhythmias and sudden cardiac death. Caucasian studies have shown a definite relationship between QTc prolongation and Cardiac Autonomic Neuropathy (CAN) in diabetic subjects.

**Objective:**

To determine the prevalence of prolonged QTc in Black diabetic individuals with CAN and to ascertain how prolonged QTc correlated with the severity of CAN among these patients.

**Methods:**

A total of 176 adult diabetic subjects were studied, 87 males and 89 females. There was a control group of non-diabetic individuals. Cardiac autonomic function was assessed using five cardiovascular autonomic function tests. CAN was diagnosed if 2 or more of these tests were abnormal. Severity of CAN was determined according to the number of abnormal tests. QTc > 0.440 was regarded as prolonged.

**Results:**

Fifty-one out of the 176 diabetic subjects (29%) had CAN. The prevalence of prolonged QTc in diabetic subjects with CAN was 12%. QTc was prolonged in 1.6% and 0.6% of diabetic individuals without CAN and controls respectively. Although QTc correlated strongly with cardiac autonomic function neuropathy, there was no definite relationship between QTc prolongation and severity of CAN.

**Conclusion:**

This study in a Black population is in agreement with the well-known relationship between QTc prolongation and CAN reported in Caucasian studies. In view of the wide variability of QTc in this study population, it is suggested that relative QTc increase may be a better indicator of CAN than a definite QTc prolongation of greater than 0.440.

## Introduction

Sudden death is probably the greatest challenge facing modern cardiology by virtue of the dramatic nature of its presentation and the number of victims claimed. Cardiac autonomic neuropathy has been identified as one of the factors predisposing to sudden cardiac death.^1^–^3^ It results from the dysfunction of the parasympathetic (vagal) or sympathetic supply to the heart or both.^4^,^5^ Autonomic cardiac neuropathy is a recognized complication in a number of diseases including diabetes mellitus.^6^ Neuropathy has been reported as the commonest chronic complication of diabetes mellitus, a disease that has been described in all races.^7^ Pop-Bussi et al^8^ reported that the prevalence of diabetic Cardiac Autonomic Neuropathy ranges from as low as 2.5% to as high as 90%. In the late 1970s, a high incidence of sudden death was reported in diabetic patients who had autonomic neuropathy.^9^,^10^ Cardio-respiratory arrest was identified as the cause of death in some cases but in the overwhelming majority the cause of death was not identified. Studies done in the 1980s showed significantly prolonged QT in diabetic patients with cardiac autonomic neuropathy.^1^,^2^ Later researchers confirmed this.^11^–^16^ The prevalence of prolonged corrected QT (QTc) in diabetic individuals with cardiac autonomic neuropathy varies from 6% to 70.5% in Caucasian studies.^1^–^16^–^19^ Prolonged QT has also been found in cardiac autonomic dysfunction due to other causes.^20^ An association between prolonged QT interval and sudden cardiac death has been found in various diseases.^21^,^22^

Prolonged QT is associated with malignant ventricular arrhythmias and this has therefore been suggested as one of the major mechanisms of sudden death in patients with diabetic autonomic neuropathy.^23^,^24^ QT prolongation in a diabetic individual is therefore of prognostic importance as this can identify those patients that are predisposed to malignant arrhythmias and sudden cardiac death.

The QT interval is the period (in seconds) necessary for the total process of depolarization and repolarization of the ventricles. It is rate-dependent and may be altered by numerous pathophysiologic and pharmacologic influences. 25 QTc is the QT corrected for heart rate.^26^ QTc values in normal subjects have been shown to be comparable in Black and White races, and in adults values greater than 0.440 are usually considered prolonged.^13^,^27^

There is little work done on its usefulness among Black diabetic subjects with cardiac autonomic neuropathy.

This study therefore was done with the objectives of determining the prevalence of prolonged QTc in Black diabetic CAN patients and to ascertain how prolonged QTccorrelates with the severity of cardiac autonomic neuropathy.

## Materials and methods

The study was done at the University of Nigeria Teaching Hospital Enugu, a designated centre of excellence for cardiovascular disease in Nigeria. Ethical clearance was obtained from the research ethical committee of this hospital and informed consent obtained from the subjects.

It was a longitudinal study, involving 176 patients drawn consecutively from diabetic patients attending the diabetic and medical outpatient clinics of the University of Nigeria Teaching Hospital Enugu.

Adult diabetic patients of both sexes aged 18–60 years were recruited in the study.

Excluded from the study were subjects having systemic conditions or on medications that may affect the cardiovascular reflex functions or QTc. These conditions include systemic hypertension, heart failure, stroke, ischaemic heart disease, uraemia, hypocalcaemia, hypokalaemia and drugs such as disopyramide, quinidine, procainamide, quinine, chloroquine, halofantrine, tricyclic anti-depressants, neuroleptics and non-sedative anti-histamins. Also excluded were subjects with ECG abnormalities of prolonged QRS duration, significant arrhythmias and ventricular hypertrophy.

There was a control group of 176 subjects, who were matched for age and sex but did not have diabetes mellitus by blood sugar criteria and also satisfied other inclusion criteria for enrolled subjects.

Standard Electrocardiograph (ECG) machine (SMELF and XDH — 3 Electrocardiograph) was used.

### Study procedure

Thorough history taking and physical examination were done for every subject with special attention to define eligibility for participation in the study.

The following investigations were done for the subjects (patients and controls): serum calcium, blood sugar, blood urea and creatinine.

### QTc measurement

i. Subjects who satisfied the clinical and laboratory inclusion criteria were enrolled for QTc measurement and cardiovascular reflex function tests. All medications other than hypoglycaemic agents were stopped for 24 – 48 hours before (ECG) testing.

The ECG was recorded at 25mm/s (and 50mm/sec for QTc measurement). A resting ECG was run to confirm that subject did not have any of the ECG exclusion criteria and to determine the R-R and QTo. The resting QTo was measured carefully according to the guidelines of Simonson et al.^25^

ii. QTc was calculated by Bazett's formula:

QTc =QTo/√R-R

QTc of >0.440 was regarded as abnormally prolonged.^13^,^27^

### Cardiovascular autonomic reflex tests

Cardiac autonomic function was assessed using five (5) cardiovascular reflex function tests.^15^,^28^,^29^ The response to each test was graded as normal or abnormal and subjects assigned cardiac autonomic function score from 0–5 based on the total number of abnormal tests. A subject was classified as having cardiac autonomic neuropathy if 2 or more tests were abnormal.^1^,^17^,^28^

**The procedures for the tests were as follows:**

a. **The resting pulse:** This was determined by examination of ECG tracing after subject had rested for at least 15 minutes. Abnormal rate was defined as 100 beats/min or more.

b. **Beat to beat heart rate (H.R) variability:** This was determined by the subject lying quietly and breathing deeply at 6 breaths/min, the difference between the minimum and maximum H.R was determined by an ECG rhythm over 1 min. Abnormal variability was defined as a difference of 10 beats/min or less.

c. **Valsalva Manoeuvre:** The subject blewinto a sphygmomanometer to maintain a pressure of 40mmHg for 15 sec under continuous ECG monitoring which continued for another 15 sec after release of strain. The ratio of the longest R-R (phase 4) after the manoeuvre to the shortest R-R (phase 2) during the manoeuvre was calculated. An abnormal response (square wave Valsalva) was defined as a ratio of 1.10 or less.

d. **Heart rate response to standing:** During continuous ECG monitoring the ratio of R-R at the 30^th^ beat after standing compared to the R-R at the 15^th^ beat (30:15) was calculated. Abnormal response was defined as a ratio of 1.00 or less.

e. **Blood pressure (BP) response to standing.** The fall in systolic BP after 1 min of standing was determined using cuff sphygmomanometer. The abnormal response was defined as a fall of 30 mmHg or more.

## Statistical analysis

The data obtained in the study was analyzed with Statistical Package for Social Sciences (SPSS) 9.0, Dbase IV and Microsoft Excel. The mean values of the QTc were compared between groups using the student's ‘t’ test. The standard deviation (SD) of the QTc within each group was determined and used to assess the variability of the results within each group. The proportions of prolonged QTc in the various groups were compared with Chi square statistic. Correlation among variables was tested by Pearson correlation for parametric variables. Regression analysis was performed with QTc as the dependent variable to reveal the relationship between QTc and cardiac autonomic function score, age of subject and duration of diabetes. Differences in results were regarded as statistically significant when p<0.05.

## Results

One hundred and seventy-six diabetic patients who met the inclusion criteria were recruited. There were 87 males and 89 females. Their ages ranged from 18 to 60 years with mean age of 46.09 ± 9.51. Their ages were matched for sex and age with 176 controls. Out of the 176 diabetic patients, 148 (84.1%) were non-insulin dependent diabetics (NIDDM) [mostly type 2] and 28 (15.9%) were insulin dependent diabetics (IDDM) [these were patients who required insulin from the onset (type 1) or required insulin following oral hypoglycaemic agents failure (type 1½)^30^,^31^.

Fifty-one (29%) of the diabetic population had Cardiac Autonomic Neuropathy (CAN) [i.e. cardiac autonomic function score of 2 or more]. Table 1 shows the frequency distribution of the diabetic subjects with CAN according to Cardiac Autonomic Function Score (CAFSCO). Of the population with CAN, 19 were males and 32 females. The mean age of diabetic subjects with CAN was 49.20 ± 7.69 years; this is significantly higher than the mean age of whole diabetic population/controls (t=2.643, P<0.01). The duration of disease in diabetic population with CAN ranged from newly diagnosed to 18 years with a mean of 6.59 ± 5.35 years. This mean is significantly longer than the mean duration of disease in diabetic individuals without CAN (3.86 ± 4.29 years) (t=3.243, p<0.01). The majority of patients (72.55%) had milder degrees of CAN (i.e. cardiac autonomic function scores [CAFSCO] of 2 & 3).

**Table 1:** Frequency distribution of diabetic subjects with cardiac autonomic neuropathy (CAN) according to cardiac autonomic function score (CAFSCO)

CAFSCO	No. of Patients	%
2	24	47.06
3	13	25.49
4	13	25.49
5	1	1.96
Total	51	100

QTc in controls ranged from 0.301 to 0.457 with mean of 0.381 ± 0.081. The mean QTc for males was 0.371 ± 0.032 which was shorter than the mean of 0.391 ± 0.108 for females. The observed difference, however, was not statistically significant (t=1.674, p>0.005).

In the whole diabetic population, QTc ranged from 0.309 to 0.504 with a mean of 0.386 ± 0.031. This mean was not significantly different from the mean QTc of controls (t=0.765, P>0.10). The mean QTc for females (0.394 ± 0.03) was significantly longer than the mean QTc for males (0.378 ± 0.031) (t=3.487, p<0.01).

Table 2 shows mean QTc according to age of the diabetic and control groups. There was no significant difference between mean QTc of patients with NIDDM (0.386 ± 0.031) and those with IDDM (0.387 ± 0.035) (t=0.141, p>0.10)

**Table 2:** Mean QT_C_ according to age of the study population (Controls/Diabetic subjects) Mean QT_c_ ± SD

Age range (yrs)	Controls	Diabetic Subjects	P-value
11 –20	0.355 ± 0.015	0.346 ± 0.003	p > 0.10
21 –30	0.329 ± 0.021	0.408 ± 0.047	p < 0.001
31–40	0.381 ± 0.163	0.379 ± 0.026	p > 0.10
41–50	0.379 ± 0.032	0.0381 ± 0.028	p > 0.10
51–60	0.390 ± 0.023	0.391 ± 0.032	p > 0.10
Total	0.381 ± 0.081	0.386 ± 0.031	p>0.10

DCAN- = Diabetic subjects without Cardiac Autonomic Neuropathy

DCAN+ = Diabetic subjects with Cardiac Autonomic Neuropathy

QTc in diabetic patients with CAN ranged from 0.374 to 0.504 with mean QTc of 0.411 ± 0.028. The mean QTc for males was 0.408 ± 0.023 while that of females was 0.412 ± 0.030.

**Figure 1:**
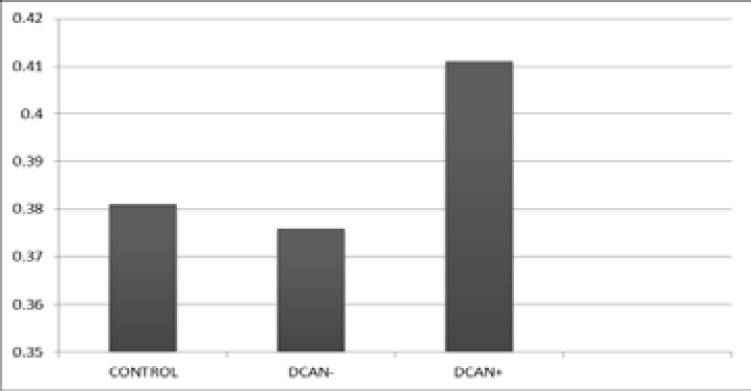
Mean qt_c_ of controls, diabetic patients with and without Cardiac autonomic neuropathy

The mean QTc of diabetic group with CAN was significantly longer than the mean QTc of diabetic subjects without CAN (0.376 ± 0.027) (t=7.60, p<0.001) and the mean QTc of controls (0.381 ± 0.081) (t=2.992, p<0.001). There is however no significant difference between mean QTc of diabetic group without CAN and controls (t=0.761, p>0.10). Figure 1 displays the mean QTc of controls, diabetic individuals with and without Cardiac Autonomic Neuropathy.

Only one subject (a female) [0.57%] out of the 176 control subjects had a QTc greater than 0.440. In the diabetic population, 8 subjects (4.55%) had prolonged QTc, six of whom had CAN. This means that 11.76% (approximately 12%) of the 51 diabetic subjects with CAN had prolonged QTc. There was significant difference in the proportions of prolonged QTc in controls, diabetics with and without CAN (X2=20.81, df=2, p<0.001). Of the six subjects that had prolonged QTc in the CAN group, one (16.67%) was a male while five (83.33%) were females. Only one subject (16.67%) out of the six subjects had severe degree of cardiac autonomic neuropathy.

Simple regression analysis showed only a weak positive correlation between QTc and age among the control group (r=0.146). There was no significant difference in the mean QTc of the various age groups of the control [Table 2; ANOVA (F-ratio=1.342, p>0.05)].

Among the diabetic subjects, correlates were sought between QTc (dependent variable) and age, duration of DM and cardiac autonomic function scores (CAFSCO). Tables 3 & 4 show that there was virtually no correlation between QTc and age (Pearson's univariate analysis r=0.080, multiple regression analysis r=0.017). Similarly, QTc did not correlate with duration of disease (univariate analysis r=0.076, multiple regression analysis r=0.068). Also there was no significant difference in the mean QTc of the various age groups of diabetics (Table 1; F=1.254, p>0.05). On the other hand, QTc was strongly correlated with CAFSCO (univariate analysis r=0.503, multiple regression analysis r=0.526). There was also a significant difference in the mean QTc of the subjects having various Cardiac Autonomic Function Scores (0–5) (F=19.306, p<0.05). However, there was virtually no significant difference in the mean QTc of CAN patients with the different CAFSCO (2–5) [F=2.814, p≈0.05]

**Table 3:** Multiple Regression Analysis of Factors that Correlate with QTC among Diabetic subjects

Unstandardized Coefficients		Standardized Coefficient			
	B	Std. Error	Beta	T	Sig.
(Constant)	0.375	0.010		36.410	0.000
* DURA	−4.486 E – 04	0.000	−0.068	−0.965	0.336
AGE	−5.713 E -05	0.000	0.017	−0.248	0.805
**CAFSCO	1.341 E – 02	0.002	0.526	7.597	0.000

* Duration of Diabetes Mellitus

** Cardiac autonomic function score

**Table 4:** Pearson Correlation: QTC, Age, Duration of DM (DURA) and Cardiac Autonomic function Score (CAFSCO) of Diabetic subjects

	QT_c_	DURA	AGE	CAFSCO
QT_c_	1.000	0.076	0.080	0.503
DURA	0.076	1.000	0.297	0.284
AGE	0.080	0.297	1.000	0.224
CAFSCO	0.503	0.284	0.224	1.000
Sig. QT_c_	−	0.158	0.144	0.000
DURA	0.158	−	0.000	0.000
AGE	0.144	0.000	−	0.001
CAFSCO	0.000	0.000	0.001	−

## Discussion

The diagnosis and assessment of severity of cardiac autonomic neuropathy was done using cardiovascular autonomic reflexes as is widely used in clinical practice and research.^15^,^17^,^28^,^29^ The use of radio-labelled analogs of norepinephrine in evaluating CAN though more direct and specific is too expensive for routine clinical assessment especially in developing countries.^32^

The prevalence of CAN (30%) in this study compares with most Caucasian values, which range from 15 to 40%.^18^,^33^,^34^

Pop-Bussi et al^8^ reported that the prevalence of CAN ranges from as low as 2.5% to as high as 90%. This wide variation may result from type of population studied and the tools used in diagnosing CAN. For example, the lower prevalence (12.5%) reported by Ofoegbu^35^ in a study with comparable clinical characteristics with present study may be explained by the use of a single cardiovascular autonomic function test (Valsava ratio) by the author as the basis for diagnosis. In the present study, the gold standard clinical autonomic testing was used.

This study showed that cardiac autonomic neuropathy affected QTc much more than other variables. QTc correlated strongly with cardiac autonomic function score, CAFSCO (r=0.526). Langen et al^36^ and Bellavere et al^2^ obtained similar strong correlation, (r=0.527) and (r=0.824) respectively. Other workers^12^,^16^,^17^ have confirmed this strong relationship between QTc and CAFSCO. QTc was consistently longer in females than males in all study groups. The mean QTc of females/males were: control 0.391 ± 0.108/0.371 ± 0.032, all diabetics 0.394 ± 0.030/0.378 ± 0.031, diabetics with CAN 0.412 ± 0.030/0.408 ± 0.023. These results are consistent with the well recognized fact that QTc is longer in females than males in both Caucasian and Black subjects.^1^,^27^,^37^ QTc gender difference reflects QTc shortening in males during adolescence.^38^ The reason for this is not obvious. In the study, the control group showed weak correlation between QTc and age (r=0.17). Simonson et al^25^ had reported that age made only a small contribution to the relationship between QT and R-R intervals. In the diabetic population age did not seem to have affected QTc significantly (r=0.080).

There was no significant difference in the QTc of the diabetic types studied (t=0.141, p>0.05). Similarly, no correlation was found between QTc and the duration of DM (r = −0.068). These findings are consistent with works by several authors.^16^,^17^,^39^ But Oka et al^12^ reported that QTc was more prolonged in patients with a long duration of DM as compared with a short duration of disease. This may be explained by the fact that higher incidence of Cardiac Autonomic Neuropathy may be found in diabetics with longer duration of disease.

The mean QTc of diabetic subjects with Cardiac Autonomic Neuropathy was significantly longer than the mean QTc of diabetics without cardiac autonomic neuropathy. This finding is in keeping with other related works.^1^,^16^,^17^

In this study, the prevalence of prolonged QTc in diabetic subjects with CAN was 12%. The prevalence of prolonged QTc in diabetics with CAN showed a wide range of 6% to 70.5% in Caucasian studies.

Tentolouris et al^17^ and Ewing and Clarke^19^ reported 6% and 14% respectively which are comparable with the result of the present study. Veglio et al^34^ reported 23%. They investigated 168 diabetics and 52 (31%) had CAN. Other clinical characteristics of their study were similar to the present study. The mean QTc of the control groups of the present study (non diabetic control 0.381 ± 0.081, DCAN-, 0.376 ± 0.027) was less than that of their control group (DCAN- 0.403 ± 0.026). This may partly explain why their prevalence was higher than that of the present study. Mahwi et al^16^ have reported that the sensitivity of prolonged QTc for detection of CAN was 20%, with specificity and positive predictive value of 98.7% and 92.3% respectively. However, Kahn et al^1^ reported a very high prevalence of 70.5%. They studied 30 diabetic individuals of whom 17 had CAN. Their sample size was rather too small and might have contributed enormously to the high figure.

The mean QTc in this study is smaller than most Caucasian studies cited. This may partly be explained by the wide variability from the mean noticed in this study (control 0.381 ± 0.081). Araoye^27^ obtained similar wide variability among healthy adult Blacks.

This raises a question as to whether the variability among Blacks is wider than that of Caucasian subjects. If this is so, it may result to smaller prevalence of prolonged QTcin Black diabetics with CAN compared with their Caucasian counterparts. Also marked differences in the mean QTc and dispersion of study populations may contribute to the wide range of the prevalence of prolonged QTc among diabetic patients with CAN. Tentolouris et al^17^ also suggested that differences in the criteria for defining diabetic cardiac autonomic neuropathy could explain the wide range.

The findings in this study suggest that relative QTc increase may be a better indicator of CAN than absolute value of QTc > 0.440.

Although QTc correlated strongly with Cardiac Autonomic Function Scores (CAFSCO) (r=0.526), there was no definite difference in mean QTc of diabetic CAN subjects with the various CAFSCO (F=2.814, P≈0.05). Out of 6 subjects that had prolonged QTc among the diabetic group with CAN, only one (16.7%) had severe degree of CAN. These findings agree with those of Kahn et al^1^ and Klupa et al^40^. Those findings asserted that QTc is not a reliable indicator of the severity of Cardiac Autonomic Neuropathy. However, other workers^3^,^17^,^20^ found prolonged QTc more with major degrees of cardiac autonomic Neuropathy. These conflicting results may support that there is no definite relationship between QTc prolongation and the severity of Cardiac Autonomic Neuropathy.

## Conclusion

There is a definite prolongation of QTc in Black diabetics with CAN in agreement with the well-known relationship between QTc prolongation and CAN. The prevalence of QTc in Diabetic Cardiac Autonomic Neuropathy in this study (12%) falls within the range obtained in Caucasian studies.

As observed in some previous studies, QTc in this study population showed a wide dispersion. It is therefore suggested that relative QTc increase may be a better indicator of CAN rather than absolute QTc prolongation of QTc > 0.440.

There may not be a definite relationship between QTc prolongation and the severity of CAN. This study recommends that all diabetics should have routine ECG monitoring for measurement of QTc. This may detect CAN and patients at risk of lethal arrhythmias and sudden cardiac death. Indiscriminate use of drugs that prolong QTc should be discouraged among diabetics.
